# Exploring Parental Perceptions and Barriers to Early Orthodontic Treatment in Children: A Mixed-Methods Study

**DOI:** 10.3390/healthcare14020180

**Published:** 2026-01-11

**Authors:** Guna Shekhar Madiraju

**Affiliations:** Faculty in Pediatric Dentistry, Department of Preventive Dental Sciences, College of Dentistry, King Faisal University, Al Ahsa 36362, Saudi Arabia; gmadiraju@kfu.edu.sa or indshe117@gmail.com

**Keywords:** early orthodontic treatment, perception, barriers, parents, mixed method design, qualitative study, child

## Abstract

Parents’ awareness, attitudes, and perceptions of barriers to orthodontic care for children significantly influence decisions regarding early orthodontic interventions. This mixed-methods study explored parents’ perceptions of their child’s orthodontic needs and examined their experiences and perceived barriers to accessing early orthodontic treatment (EOT) among children aged 6–12 years. **Methods**: Quantitative data were collected using a 12-item validated questionnaire, while qualitative insights were obtained through structured interviews and analyzed thematically. **Results**: Parents’ perception of their child’s orthodontic needs was significantly associated with their attitude toward seeking consultation or treatment (*p* < 0.001). Among parents who sought consultation, only 38.7% had initiated the required orthodontic treatment. The most frequently reported barriers were high cost (32.1%), long appointment wait times (30.6%), and lack of insurance coverage (22.5%). Thematic analysis revealed four major barriers: financial, structural, cognitive, and psychological. **Conclusions**: These findings highlight critical challenges to accessing EOT for children, including affordability, long waiting times, limited parental awareness, and inadequate, timely referrals. Addressing these challenges through combined efforts at both the individual and community levels could significantly enhance the uptake of early orthodontic services in children.

## 1. Introduction

Early orthodontic intervention during the mixed dentition stage is crucial for identifying and addressing potential orthodontic issues in children. Timely intervention can reduce the need for more complex treatment in the future and also enhance oral health over the long term. In Saudi Arabia, despite the integration of orthodontic services within the public healthcare system, a substantial proportion of children continue to experience unmet orthodontic treatment needs, particularly during the mixed dentition stage [1]. These gaps are influenced by parental awareness and referral practices of oral healthcare providers [2,3,4,5]. Studies have shown that parents’ awareness and attitudes toward seeking early orthodontic care are influenced by a variety of factors, including socioeconomic, emotional and cultural considerations, which contribute to the disparity in access to orthodontic care [5,6,7].

Understanding the barriers to accessing orthodontic care is essential not only for improving service accessibility and equity but also for facilitating the effective integration of orthodontic services into the broader healthcare framework, thereby enhancing oral health outcomes. Most studies on barriers to healthcare access have relied primarily on quantitative methods [1,2,6], providing limited insight into the contextual, psychological, and experiential factors influencing parental decision-making. Moreover, evidence focusing specifically on early orthodontic treatment during the mixed dentition stage remains scarce. This study addresses this gap by employing a convergent mixed-methods approach to comprehensively explore parental perceptions and perceived barriers to early orthodontic treatment (EOT) in children within the Saudi Arabian healthcare context. This focus aligns with one of the core objectives of Saudi Arabia’s Vision 2030 initiative, which emphasizes the prioritization of the healthcare sector, including expanding healthcare coverage [8].

## 2. Materials and Methods

### 2.1. Study Design

**Figure 1:**
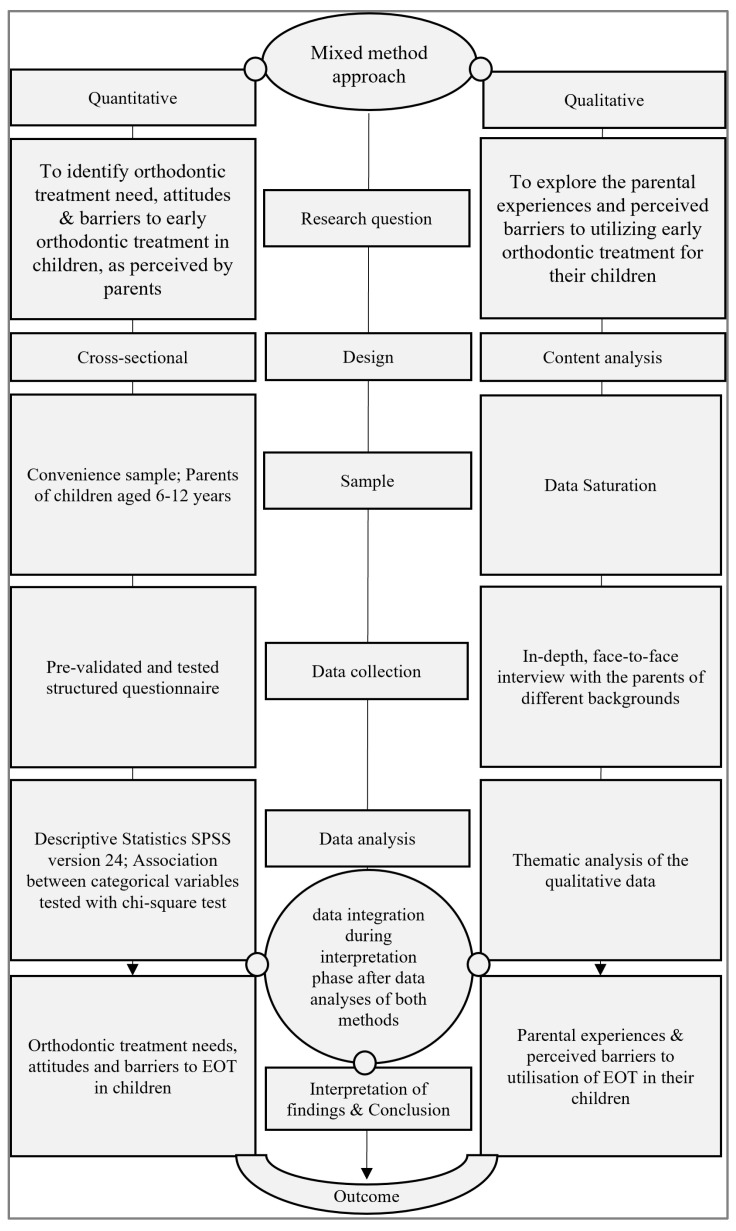
Flowchart of mixed-methods approach.

This study employed a convergent mixed-methods design [9] to explore the parents’ attitudes and perceived barriers to EOT during the mixed dentition stage in children from the eastern province region of Saudi Arabia. In the first phase, the study used a structured questionnaire (quantitative) to evaluate the parents’ attitudes and perceived barriers to utilization of EOT in children, through a set of 12 questions, while in the second phase, a qualitative approach of in-depth, face-to-face interviews was conducted to comprehensively assess the perceived barriers and experiences, as perceived by parents. The study design flowchart is presented in Figure 1.

#### 2.1.1. Quantitative Phase

Parents of children aged 6–12 years who visited the university dental hospital seeking pediatric dental care services were invited to participate in the study, during the period from November 2024 to June 2025. The study design and protocols were reviewed and approved by the institutional ethics and review board.

This study used convenience sampling, and a minimum sample size of 384 participants was required based on a confidence level of 95% and a 5% margin of error. The inclusion criteria were parents accompanying 6–12-year-old pediatric dental patients of Saudi origin and who had at least one previous dental screening visit. Parents of children with systemic illness and those parents with limited reading and writing skills were excluded.

Data collection:

The data collection tool included a validated and tested questionnaire adopted from previous studies in the literature [10,11]. The questionnaire was slightly modified and translated into the local Arabic dialect by a bilingual (Arabic–English) healthcare specialist, and its accuracy was reviewed and validated using standard forward and back translation procedures. It was then pilot-tested on twenty parents not included in the main study, confirming its clarity and suitability without requiring further modifications. A QR code was created to make it easier for parents to digitally fill out the questionnaire themselves, without any time constraints. Prior to accessing the questionnaire, participants were required to provide digital informed consent, and those who declined consent were automatically exited from the survey. Internal consistency of the questionnaire was evaluated using Cronbach’s alpha. The instrument showed good overall reliability (α = 0.82). Reliability across domains was acceptable, with alpha coefficients of 0.79 for parental perception (Q7–Q9), 0.77 for attitude (Q10–Q11), and 0.81 for perceived barriers (Q12). These results indicate satisfactory internal consistency of the adapted and translated questionnaire in the study population.

The questionnaire consisted of 12 items divided into two parts, with the first part focusing on the demographic data of participants (Q1–Q6), including parents’ age, gender, highest educational level, family monthly income, number of children, and personal exposure to any previous orthodontic care. The remaining questions assessed the parental perception of their child’s orthodontic treatment needs (Q7–Q9), parental attitudes (Q10–Q11), and perceived barriers to utilization of EOT for their children (Q12) (Supplementary File S1). Study participants were allowed to select one or more items to indicate perceived orthodontic treatment needs (Q9) and probable barriers to the treatment (Q12) based on their perceptions. The outcomes were the parental attitudes and perceived barriers to EOT in children in the mixed dentition stage. The exposures were parental age, gender, monthly income, educational level, personal history of orthodontic treatment, and number of children.

For analytical purposes, ‘Orthodontic consultation’ was operationally defined as a parental affirmative response to having previously consulted a dentist or orthodontist for orthodontic evaluation of their child (Q10). ‘Treatment initiation’ was defined as an affirmative response indicating that the child had commenced the recommended orthodontic treatment following consultation (Q11).

#### 2.1.2. Qualitative Phase

The reporting of the qualitative study was in line with the Declaration of Helsinki and followed the Consolidated Criteria for Reporting Qualitative (COREQ) research checklist [12].

Data collection:

Purposive sampling was used, and participants with different educational backgrounds were recruited to ensure diversity in parental perspectives, as educational level is known to influence health-related attitudes and decision-making [7]. Data were collected through semi-structured interviews, conducted in a noise-free section of the clinical setting. Each interview was conducted by an experienced clinician trained in qualitative research to ensure proper data collection in line with the study objectives. The interviews were carried out in Arabic and/or English language, with a bilingual (Arabic–English) healthcare specialist (not part of the study) serving as a translator when needed. Prior to the sessions, a pilot trial was carried out on five parents to test the questions for clarity and consistency, including the appropriateness of the language and guiding questions. The results of the pilot study have not been included in the study. The study objectives were explained to the participants, and rapport was established prior to the start of the interview. A written informed consent form was distributed to all participants and signed before the interviews were conducted. Participation in the interview was voluntary, and participants could withdraw from the study at any time.

The participants were interviewed with mainly open-ended questions (one-on-one interview) to understand their perceptions on barriers to utilization of EOT in children. The interview approach was flexible, including the selection and sequencing of questions based on the participants’ responses to previous questions. Each interview lasted approximately 20–30 min and was concluded upon completion of gathering the required information. Participants were allowed to share their experiences, but the conversations could not be audio-recorded due to cultural constraints and personal preferences in the local community. Detailed notes were taken where possible during the interviews, and after each interview, the notes were reviewed with the participant to check if they represented their thoughts and opinions. These interviews among parents were conducted until the data saturation point was reached. Data saturation was assessed iteratively during data collection, and interviews were conducted until no new themes, categories, or conceptual insights emerged from the data. Saturation was achieved after 20 interviews, as subsequent interviews yielded repetitive information without the emergence of new codes or themes. Participants were acknowledged and apprised of their participation, and no incentive was provided.

#### 2.1.3. Statistical Analysis

Data were statistically analyzed using Statistical Package for the Social Sciences software (SPSS^®^ for Windows, version 24: IBM Corp., Armonk, NY, USA). Categorical variables, presented as percentages and frequency distributions, were assessed using the Chi-square test. A level of significance was established at less than 0.05.

Thematic content analysis of the qualitative interview data was conducted manually by a single investigator using an inductive approach. Data from the interview transcripts were translated into English and thoroughly reviewed. Care was taken to ensure no personal information was included in the translated record, which was then assigned an alphanumeric reference code to ensure confidentiality. The investigator read the transcripts multiple times to gain familiarity with the data and generated initial codes relevant to the research objectives. Codes with similar characteristics were grouped into broader categories, and potential emerging themes were identified. These themes were refined through iterative analysis to ensure they accurately reflected participant responses. The themes were then finally refined, summarized, and the study findings were documented. To enhance analytical rigor and address the potential for subjectivity inherent in using a single coder, intra-coder reliability was assessed. A randomly selected subset comprising 25% of the transcripts was re-coded by the same investigator after a four-week interval. The consistency of coding was reviewed, and the intra-coder agreement exceeded 90%, indicating a high level of coding stability over time.

## 3. Results

### 3.1. Quantitative Results

A total of 470 parents consented and participated in completing the questionnaire, among whom 53.4% were females and 46.6% were males. The mean age of the children was 8.8 ± 1.45 years (Mean ± SD). The distribution of the responses of the study sample to the survey questions is shown in Table 1. In terms of age group, the majority of them (53.2%) were aged below 30 years. Most respondents (53.8%) had completed at least a secondary/high school education, followed by those with a college/university level education (35.3%). In total, 269 (57.2%) of the parents thought that their children’s teeth would have a significant impact on their personality, while 320 (68.1%) parents thought that their children had a problem with alignment/position of their teeth and/or jaws. Protruded upper teeth (28.8%) followed by missing (22.8%) and crowded teeth (18.1%) were among the most commonly perceived orthodontic problems in the children. Around 64.3% (*n* = 302) of the total parents sought advice regarding their child’s consultation with a dentist or orthodontist, but only 39.7% (*n* = 120) among them had initiated the required orthodontic treatment. With regard to perceived barriers to utilization of EOT in children, high treatment cost (32.1%) was the most common reason not to pursue early orthodontic care, followed by the long waiting list in primary healthcare centres (30.6%) and lack of insurance coverage (22.5%) for the orthodontic treatment in children [Table 1]. The association between parental perception of their child’s orthodontic needs and their attitudes toward EOT (*p* < 0.001) was statistically significant.

The association between the number of children in a family and parental perception of their child’s orthodontic needs (as measured by Q7–Q9) and parental attitudes toward EOT (as measured by Q10–Q11) was found to be statistically significant (*p* < 0.001). Parents with a higher number of children tended to show a lower perception of their child’s orthodontic treatment need and less favorable attitudes toward EOT for their children. Parental age, education, family monthly income and the number of children (*p* < 0.05) had a significant difference with respect to parental perceptions of their child’s orthodontic treatment need, and attitudes toward EOT, while gender and personal history of orthodontic treatment showed no such statistical significance (*p* > 0.05) except for attitude (Q10) to consult a dentist or orthodontist seeking orthodontic evaluation (*p* < 0.001) [Table 2 and Table 3].

### 3.2. Qualitative Results

A total of 20 parents were interviewed (9 females and 11 males) at the point of data saturation. All the participants were of Saudi origin in the age group 28–41 years. They were asked to describe the perceived difficulties or barriers to seeking EOT for their children. For confidentiality purposes, quotes are anonymized and interviews labelled as P1-P20. Four main themes emerged from the in-depth interviews, and representative participant quotes were purposefully selected to illustrate key themes and sub-themes.

Financial barriers:

Most parents consistently highlighted the financial burden associated with orthodontic care as one of the most crucial barriers to seeking orthodontic treatment for their child. Affordability (subtheme 1) was a key barrier due to high treatment costs. For many, the costs of treatment are prohibitive, especially considering that basic dental services are often provided for free through the public healthcare system in Saudi Arabia. As one parent stated:

*“Most basic dental treatments are provided to us free of cost at primary healthcare centres, but orthodontic care is so expensive that it’s difficult for us to afford it.”*

*(P2)*

Another parent who felt orthodontic treatment was costly addressed the issue by managing to pay the fee in instalments.

*“Orthodontic treatments at private clinics are very expensive. As a parent I want to ensure my child receives best care possible without the burden of financial stress. To manage the costs, we request the dentist to reduce the fee sometimes or allow the option to pay in instalments.”*

*(P4)*

A related concern was the lack of insurance coverage (subtheme 2) for orthodontic treatment, which was found to play a large role in the decision-making whether to seek, delay or avoid orthodontic services for their child. Some parents noted that even when they had medical insurance coverage, it often excluded orthodontic procedures, leaving families to bear most of the costs.

*“There is no insurance coverage provided for my child’s orthodontic treatment through our medical insurance. Without coverage, we’re left with a heavy financial burden which really affects our family expenses. It’s hard to understand why something so important for their health is not covered.”*

*(P7)*

Structural barriers:

Although basic dental treatments are provided in primary health centres, some participants believe that service availability (subtheme 1) of orthodontic care in selected locations within the region was satisfactory. Alternatively, they are provided mostly by the private sector. Parents who relied on the public health system, i.e., primary health centers, frequently encountered long appointment wait times (subtheme 2) for orthodontic consultations and/or treatment. These delays often discouraged parents from pursuing orthodontic care for their children.

*“Orthodontic care in primary health care centres comes with long appointment wait times. We had to wait months just to get an appointment. When I requested to make an appointment for my child, they told me the next available slot was eight months away, and so we….”*

*(P3)*

Cognitive barriers:

Parents demonstrated limited awareness (subtheme 1: Lack of awareness) of the importance of early orthodontic intervention in children. Some participants assumed that orthodontic care was primarily for cosmetic reasons or correcting teeth alignment problems during adolescence. One parent stated:

*“I didn’t know my child needed orthodontic care at such a young age. To be honest, I thought braces or other orthodontic appliances were something only teenagers or adults required, not children.”*

*(P9)*

Some parents had a common opinion that the baby teeth would eventually fall off and new teeth would come in their place, leading them to assume that orthodontic care was not urgent. One parent remarked that:

*“My child’s teeth doesn’t need orthodontic treatment with braces at this young age. Those baby teeth will fall off anyway and another new set of teeth will come”.*

*(P18)*

Another parent informed the interviewer that the last time they had sought dental treatment for their child, the primary dental care provider had informed the parents that they could delay the orthodontic or corrective treatment till all the primary teeth fall off and the complete set of permanent teeth has erupted. Lack of timely referral and clear guidance (subtheme 2) for orthodontic care from primary dental care providers was expressed.

*“My dentist never mentioned that my child might need to see an orthodontist. So, i assumed everything was fine with her teeth. How was I supposed to know that these issues could go unnoticed until they become more serious”?*

*(P5)*

*“When we took our child to the dentist for a check-up, the dentist advised us to wait on orthodontic treatment until all the baby teeth fall out and the permanent ones come in. He said it was better to wait and see how the permanent teeth adjust before starting any treatment with braces. While that advice made sense at the time, now when we look at the cost and the need for treatment, it’s becoming a concern for both my child’s health and our family’s finances.”*

*(P11)*

Psychological barriers:

This theme included the dimension of an emotional impact on the parental perceptions. Parents shared concerns about their child’s fear of dental procedures and their own anxieties (subtheme 1: fear and anxiety) often compounded each other, leading to delayed decision-making or avoidance in seeking care.

*“My child is already scared of the dentist. The thought of taking him to an orthodontist for even more appointments makes both of us feel anxious.”*

*(P14)*

Some parents expressed/shared concerns about the potential discomfort or inconvenience to school activities and daily routine (subtheme 2: Impact on Lifestyle and Routine) due to the restrictions involved with orthodontic appliance treatment, rather than on financial constraints (financial cost of treatment).

*“I’m not concerned about the cost of the treatment, but I do worry that some types of orthodontic appliances might interfere with my child’s school activities and daily routine.”*

*(P10)*

## 4. Discussion

This mixed-methods study provides a comprehensive examination of parental perceptions and barriers to early orthodontic treatment among children in the Eastern Province of Saudi Arabia. Quantitative findings revealed significant associations between parental sociodemographic factors and attitudes toward early orthodontic care, while qualitative insights identified financial, structural, cognitive, and psychological barriers that influence parental decision-making. Together, these findings offer an integrated understanding of the multifaceted challenges affecting timely orthodontic care for children. By employing a mixed-methods design, this study integrated quantitative breadth with qualitative depth to provide a comprehensive and nuanced understanding of parental perceptions and the reasons for not utilizing available orthodontic services for their children. Triangulating findings from both methodological strands allowed for the identification of areas of convergence and divergence during interpretation, thereby enriching the overall contextual understanding of parents’ attitudes and perceived barriers. To the best of our knowledge, this is the first study to use a mixed-methods approach to examine parental perceptions of barriers to early orthodontic care in children.

### 4.1. Parental Perceptions and Demographic Correlates

The quantitative results demonstrated significant variability in parental perceptions based on age, educational level, family income, and number of children in the family. In contrast, gender and personal history of orthodontic treatment were not found to influence parental perceptions. Additionally, younger parents exhibited greater awareness of early orthodontic care and were more proactive in seeking appropriate treatment, which is similar to that reported by Eshky et al. [13]. Contrary to this, Assery et al. [14] and Di Spirito et al. [11] found that parents’ older age was associated with a greater awareness of orthodontic needs.

In the present study, parents with a higher number of children tended to perceive a lower need for orthodontic treatment for their child and demonstrated less favorable attitudes toward early orthodontic treatment. In contrast, Aldweesh et al. [6] reported that a greater number of children per family was associated with an increased parental perception of orthodontic treatment need. Meanwhile, García et al. [15] reported no statistically significant association between the number of children in a household and the likelihood of initiating orthodontic consultations, aligning partially with the present findings.

The association between parental awareness of psychological impact and their attitudes toward early orthodontic care was significant in the present study. This implies that parents who acknowledged the potential emotional or social consequences of orthodontic problems were more likely to seek orthodontic advice for their children, a finding which was consistent with previous studies [6,11]. It is important to note that the question regarding whether a child’s teeth could significantly impact their personality (Q7) may be interpreted differently depending on the child’s dental condition. Parents of children with severe malocclusion or noticeable orthodontic issues may perceive a stronger potential psychological impact, whereas parents of children with well-aligned teeth may interpret the question more hypothetically, recognizing that dental appearance alone does not determine personality. Therefore, the observed association between parental awareness of psychological impact and attitudes toward early orthodontic care should be considered in the context of this potential variability in perception.

A notable percentage of parents (68.1%) perceived that their children had orthodontic problems, with protruding upper teeth, missing and crowded teeth being the most commonly identified issues. This reflects the growing awareness among parents regarding orthodontic issues. However, only 39.7% of parents, in the present study, had initiated the required treatment for their children, from among those who had sought orthodontic consultation, a finding which was lower than that reported by Di Spirito et al. [11] and Aldweesh et al. [6]. Although a majority of parents recognized orthodontic problems in their children, treatment initiation remained low, suggesting that awareness alone does not translate into service utilization. This discrepancy underscores the influence of systemic and psychological barriers that extend beyond knowledge, highlighting the need for structural and policy-level interventions. Among the barriers perceived by parents, high treatment cost, long appointment waiting lists, and lack of insurance coverage for the orthodontic treatment were noted as the prime reasons for not pursuing early orthodontic treatment. These findings are in agreement with previous studies in the literature [2,13,16,17,18]. Variations between the present findings and those reported in earlier studies may be attributed to differences in study design, population characteristics, healthcare system structure, and cultural expectations. Additionally, regional disparities in access to public orthodontic services and referral practices within primary dental care may contribute to the observed variations in treatment uptake and parental attitudes across studies.

### 4.2. Qualitative Findings: Barriers to Early Orthodontic Care

Four primary themes emerged from the thematic analysis, which included financial, structural, cognitive, and psychological barriers. These themes align with the Healthcare Access Barriers (HCAB) model of Carillo et al. [19], as well as the updated Healthcare Access Barriers for Vulnerable Populations (HABVP) model proposed by George et al. [20], which includes psychological barriers as a distinct dimension, particularly relevant in the context of healthcare access for children.

Financial barriers emerged as the most commonly cited barrier in this study. This finding is consistent with previous research across both Arab and global populations, where the high cost of orthodontic care is often identified as a major deterrent to seeking or initiating treatment [2,13,16,18,21]. The similarity in financial barriers across Saudi Arabia and other regions can be attributed to the rising costs of medical care, limited or absent health insurance coverage for orthodontic treatment, and reliance on private dental clinics. Many parents reported that orthodontic treatment in children, involving either fixed or removable appliances, was considerably more expensive in private clinical settings than services provided through the public healthcare system in Saudi Arabia. This variability in institutional costs between public and private sectors may shape parental perceptions of affordability and influence decisions regarding access to orthodontic care. Moreover, the lack of insurance coverage for orthodontic procedures was a consistent concern shared by many parents. This exclusion contributed to a perception of orthodontic care as being inaccessible, particularly for families from low- to middle-income backgrounds. Similar concerns regarding insurance limitations have been reported by other authors [18,22]. Some parents (P4), despite acknowledging the high costs, attempted to manage these challenges through instalment payment options or fee negotiations, highlighting the severity of the financial barrier.

The structural barriers highlighted in this study by parents relying on public health systems included long waiting times for appointments, which reflect systemic or structural challenges in oral healthcare delivery. Parents had frequently reported waiting several months for an initial appointment or follow-up treatment (P3). These observations are congruent with previous studies [22,23,24]. The similarity in structural barriers across regions may be attributed to a potential shortage of orthodontic specialists and/or their concentration in urban centres, as reported by Felemban et al. [18] and Alharbi and Taju [25] in their studies. However, in this study, none of the interviewed parents explicitly identified a shortage of orthodontists as a barrier to seeking treatment.

Lack of awareness regarding the importance and timing of early orthodontic care was another prominent barrier identified in this study. Many parents perceived orthodontic care as necessary only during adolescence or adulthood for cosmetic purposes (P9). Misconceptions about primary teeth and delayed or inconsistent referral practices from primary care providers could have further contributed to reduced uptake of early orthodontic treatment. Similar gaps in parental awareness have been reported in previous studies [1,20]. Several studies have also indicated inadequate referral practices and knowledge among general dentists [2,3,25,26] and physicians [27,28]. This could possibly be due to the lack of targeted educational campaigns for parents and insufficient professional development programs for general dental practitioners aimed at raising awareness about the benefits of early orthodontic intervention.

The psychological barriers identified included fear or anxiety related to dental visits and concerns about the potential impact of orthodontic treatment or appliances on children’s daily routines. Parents expressed worries about discomfort, lifestyle disruptions and school-related challenges. These emotional or social considerations often outweighed financial factors and perceived healthcare needs, suggesting that psychological factors play a significant role in treatment-decision making. Lin et al. [29] indicated that cost was not the deciding factor, but rather a consideration prior to seeking orthodontic treatment. While cultural discrimination has been reported as a barrier in other populations [20], no such concerns were identified in the present study.

It should be noted that the qualitative findings are based on a small purposive sample of 20 parents, and the themes identified should be interpreted as exploratory and inductive. While these insights highlight potential barriers to early orthodontic care, they may not fully represent the wider population. No formal statistical analyses were applied to the qualitative data, and therefore these findings should be considered as preliminary evidence to guide future research rather than definitive conclusions for policy or practice.

### 4.3. Implications for Practice and Policy

Orthodontic care in Saudi Arabia is effectively integrated into the existing public healthcare system, with a well-established referral process and efficient scheduling system, as the majority of the population relies on public healthcare services. However, long waiting times for orthodontic treatment remain a significant challenge, which could be due to the prioritization of services for patients with more urgent needs owing to the high prevalence of unmet orthodontic care, thereby posing challenges to the public healthcare system. Potential solutions to overcome these structural barriers could include increasing the number of orthodontic specialists through public–private partnerships, implementing targeted dental public health education campaigns, and adopting patient-centered approaches to reduce anxiety. Providing parents and children with clear information on the benefits of orthodontic treatment and the lifestyle adjustments required could further support informed decisions. Furthermore, policy reforms aimed at subsidizing orthodontic treatment costs and expanding insurance coverage could significantly improve access to early orthodontic care. This could help reduce psychological and financial burdens on both the families involved and the publicly funded healthcare system in Saudi Arabia.

### 4.4. Strengths and Limitations

One of the key strengths of this study was the mixed-methods approach, which provided a deeper understanding of the perceived barriers, adding contextual richness and enhanced interpretation of the research findings. Secondly, the purposive sampling based on educational diversity allowed the study to capture a wide range of parental perspectives, and although the sample size was small, data saturation was assessed iteratively during the qualitative phase, which strengthens the credibility of the study findings. The study specifically addressed the barriers within the Saudi Arabian healthcare context, making the findings relevant for policymakers and healthcare providers in the region.

While triangulating quantitative and qualitative findings enhances contextual understanding, some limitations should be acknowledged. Differences in sample sizes between the quantitative and qualitative phases may limit direct comparisons. Moreover, qualitative responses are inherently subjective and, despite the purposive selection of a small sample with varied educational backgrounds to capture a range of perspectives, the findings remain exploratory and may not adequately represent the broader population. Additionally, the study population consisted of parents of children attending a dental healthcare center in a university setting. These factors may affect the generalizability and underestimate access barriers in the wider population. In addition, the study focused on parental perceptions and did not include the perspectives of oral healthcare providers (e.g., orthodontists or general dentists) regarding barriers to early orthodontic care, which could have provided further insights into the challenges. Another limitation of the qualitative component was the inability to audio-record interviews due to cultural sensitivities and participant preferences. Although detailed notes were taken and confirmed with participants immediately after each interview, the absence of audio recordings may have limited the completeness of the data. Furthermore, the potential for response bias among parents should not be overlooked. Further studies involving wider population groups might provide more comprehensive insights into the barriers to early orthodontic care and contribute to more targeted and effective interventions to enhance the accessibility of orthodontic care and ensure better oral health outcomes for children.

## 5. Conclusions

This mixed-methods study synthesized quantitative and qualitative evidence and demonstrated that access to early orthodontic care for children is influenced by a complex interaction of parental perceptions and systemic healthcare barriers rather than awareness alone. The findings indicate that financial constraints, service availability, referral practices, and psychological concerns collectively influence parental decision-making and contribute to delayed care. These findings underscore the need for coordinated, system-level strategies that enhance referral pathways, expand insurance coverage, improve service capacity, and address psychological barriers. Future research should explore the effectiveness of policy-driven interventions aimed at improving access to early orthodontic care and assess their long-term impact on pediatric oral health outcomes.

## Figures and Tables

**Table 1:** Demographic data and distribution of the parental responses to the questionnaire.

Question	Answer Options	Response Count	Response %
Q1. Relation to the child (*n* = 470)	Mother	251	53.4
	Father	219	46.6
Q2. Parent Age (*n* = 470)	<30	250	53.2
	30–40	151	32.1
	>40	69	14.7
Q3. Education Level (*n* = 470)	Primary School or less	51	10.9
	Secondary/High School	253	53.8
	College/University	166	35.3
Q4. Monthly Income (*n* = 470)	<5000	68	14.5
	5000–15,000	236	50.2
	>15,000	166	35.3
Q5. No. of Children (*n* = 470)	1	148	31.5
	2	104	22.1
	3 or more	218	46.4
Q6. Personal History of Orthodontic Treatment (*n* = 470)	Yes	172	36.6
	No	298	63.4
Q7. Do you think that your child’s teeth would ever have a significant impact on his/her personality? (*n* = 470)	Yes	269	57.2
	No	201	42.8
Q8. Do you think your child has any problems with the Teeth alignment/position or skeletal problems? (*n* = 470)	Yes	320	68.1
	No	150	31.9
Q9. If Yes, what problem is it? Choose one or more answers. (*n* = 320)	Spacing between teeth	35	10.9
	Crowded anterior teeth	58	18.1
	Protruded upper teeth	92	28.8
	Extra teeth	8	2.5
	Missing teeth	73	22.8
	Incorrect teeth position	25	7.8
	Facial Asymmetry	19	5.9
	Protruding mandible	18	5.6
	Protruded upper Jaw	27	8.4
Q10. Have you ever consulted a dentist or orthodontist seeking orthodontic evaluation for child? (*n* = 470)	Yes	302	64.3
	No	168	35.7
Q11. If Yes to Q10, did your child receive the required orthodontic treatment? (*n* = 302)	Yes	120	39.7
	No	182	60.3
Q12. Perceived barriers to orthodontic care in children. Choose one or more answers.	Dental fear/Anxiety	34	7.2
	Treatment cost	151	32.1
	Lack of insurance coverage	106	22.5
	Lack of knowledge	70	14.9
	Age (too young)	33	7.02
	Unaesthetic appearance	16	3.4
	Long Treatment duration	17	3.6
	Long appointment wait list	144	30.6

**Table 2:** Association between study variables and parental perception of orthodontic needs.

Question	Response	Q7. Do You Think That Your Child’s Teeth Would Ever Have a Significant Impact on His/Her Personality? (*n* = 470)			Q8. Do You Think Your Child Has Any Problems with the Teeth Alignment/Position or Skeletal Problems? (*n* = 320)		
		Yes	No	*p*-Value *	Yes	No	*p*-Value *
Q1. Relation to the child	Mother	147	104	0.532	165	86	0.242
	Father	122	97		155	64	
Q2. Parent Age	<30	168	82	0.000	218	32	0.000
	30–40	99	52		100	51	
	>40	02	67		02	67	
Q3. Education Level	Primary School	17	34	0.000	17	34	0.000
	Secondary/High School	118	135		185	68	
	College/University	134	32		118	48	
Q4. Monthly Income	<5000	51	17	0.000	68	00	0.000
	5000–15,000	152	84		168	68	
	>15,000	66	100		84	82	
Q5. No. of Children	1	116	32	0.000	133	15	0.000
	2	70	34		103	01	
	3 or more	83	135		84	134	
Q6. Personal History of Orthodontic Treatment	Yes	95	77	0.505	116	56	0.820
	No	174	124		204	94	

* Pearson Chi-Square; *p* < 0.05, significant association.

**Table 3:** Association between study variables and parental attitudes to early orthodontic treatment.

Question	Response	Q10. Have You Ever Consulted a Dentist or Orthodontist Seeking Orthodontic Evaluation for Child (*n* = 470)			Q11. Did Your Child Receive the Required Orthodontic Treatment (*n* = 302)		
		Yes	No	*p*-Value *	Yes	No	*p*-Value *
Q1. Relation to the child	Mother	142	109	0.000	88	54	0.568
	Father	160	59		94	66	
Q2. Parent Age	<30	182	68	0.000	148	34	0.000
	30–40	117	34		33	84	
	>40	03	66		01	02	
Q3. Education Level	Primary School	17	34	0.000	00	17	0.000
	Secondary/High School	151	102		51	100	
	College/University	134	32		131	03	
Q4. Monthly Income	<5000	0	68	0.000	00	00	0.000
	5000–15,000	168	68		83	85	
	>15,000	134	32		99	35	
Q5. No. of Children	1	148	0	0.000	147	01	0.000
	2	36	68		01	35	
	3 or more	118	100		34	84	
Q6. Personal History of Orthodontic Treatment	Yes	133	39	0.000	75	58	0.222
	No	169	129		107	62	

* Pearson Chi-Square; *p* < 0.05, significant association.

## Data Availability

The dataset used and analyzed in this study is available from the corresponding author. The data are not publicly available due to ethical restrictions and institutional policies.

## Supplementary Materials

The following supporting information can be downloaded at: https://www.mdpi.com/article/10.3390/healthcare14020180/s1. Supplementary File S1: Annexure A (Study Questionnaire).

## References

[1] Madiraju G.S., Almugla Y.M., Mohan R., Alnasser B.M. (2024). An epidemiological study on early orthodontic treatment need among eastern Saudi Arabian children in the mixed dentition stage. Sci. Rep..

[2] Alshammari A.K., Alanazi A., Al-Swedani H., Khan M., Ahmad S., Haque S., Khan S. (2023). Knowledge and perception of orthodontic treatment among general and non-orthodontic dental specialists: A comparative study. Healthcare.

[3] Nobre R., Pozza D.H. (2023). Parental influence in orthodontic treatment: A systematic review. Med. Pharm. Rep..

[4] Brumini M., Slaj M., Katic V., Pavlic A., Trinajstic Zrinski M., Spalj S. (2020). Parental influence is the most important predictor of child’s orthodontic treatment demand in a preadolescent age. Odontology.

[5] Tuncer C., Canigur Bavbek N., Balos Tuncer B., Ayhan Bani A., Çelik B. (2015). How Do Patients and Parents Decide for Orthodontic Treatment-Effects of Malocclusion, Personal Expectations, Education and Media. J. Clin. Pediatr. Dent..

[6] Aldweesh A.H., Ben Gassem A.A., AlShehri B.M., AlTowaijri A.A., Albarakati S.F. (2022). Parents’ Awareness of Early Orthodontic Consultation: A Cross-Sectional Study. Int. J. Environ. Res. Public Health.

[7] Moshkelgosha V., Kazemi M., Pakshir H., Safari R. (2016). Parental Knowledge and Attitude towards Early Orthodontic Treatment for Their Primary School Children. Iran. J. Orthod..

[8] Mani Z.A., Goniewicz K. (2024). Transforming Healthcare in Saudi Arabia: A Comprehensive Evaluation of Vision 2030′s Impact. Sustainability.

[9] Creswell J.W., Clark V.L.P. (2018). Designing and Conducting Mixed Methods Research.

[10] Hassan F., Shafiq U., Mahmood A. (2016). Parental Motivation for Orthodontic Consultation during Their Child’s Mixed Dentition Phase: A Questionnaire Study. Pak. Orthod. J..

[11] Di Spirito F., Cannatà D., Schettino V., Galdi M., Bucci R., Martina S. (2024). Perceived Orthodontic Needs and Attitudes towards Early Evaluation and Interventions: A Survey-Based Study among parents of Italian School-Aged Children. Clin. Pract..

[12] Tong A., Sainsbury P., Craig J. (2007). Consolidated criteria for reporting qualitative research (COREQ): A 32-item checklist for interviews and focus groups. Int. J. Qual. Health Care.

[13] Eshky R.T., Althagafi N.M., Alsaati B.H., Alharbi R.A., Kassim S.A., Alsharif A.T. (2019). Self-Perception Of Malocclusion And Barriers To Orthodontic Care: A Cross-Sectional Study In Al-Madinah, Saudi Arabia. Patient Prefer. Adherence.

[14] Assery M.K.A. (2020). Knowledge, attitude, and practice regarding prosthodontic rehabilitation and factors affecting the patients visiting private clinics in Riyadh, Saudi Arabia: A crosssectional study. J. Fam. Med. Prim. Care.

[15] García E., Santos L., Borrell C., Aura J.I., Marqués L. (2025). Parental Perceptions of Early Interceptive Orthodontic Intervention in Children and Adolescents: A Cross-Sectional Study. Eur. J. Paediatr. Dent..

[16] Paes da Silva S., Pitchika V., Baumert U., Wehrbein H., Schwestka-Polly R., Drescher D., Kühnisch J., Wichelhaus A. (2020). Oral health-related quality of life in orthodontics: A cross-sectional multicentre study on patients in orthodontic treatment. Eur. J. Orthod..

[17] Zulkiffili A.M., Roslan L.H., Azrin N.H.I., Azmi N.N., Manivannan P.C., Yap Y.H. (2023). Assessment of self-perceived malocclusion and barriers to orthodontic treatment: A cross-sectional study. J. Orthod. Sci..

[18] Felemban O.M., Alharabi N.T., AAlamoudi R.A., Alturki G.A., Helal N.M. (2022). Factors influencing the desire for orthodontic treatment among patients and parents in Saudi Arabia: A cross-sectional study. J. Orthod. Sci..

[19] Carrillo J.E., Carrillo V.A., Perez H.R., Salas-Lopez D., Natale-Pereira A., Byron A.T. (2011). Defining and targeting health care access barriers. J. Health Care Poor Underserved.

[20] George S., Daniels K., Fioratou E. (2018). A qualitative study into the perceived barriers of accessing healthcare among a vulnerable population involved with a community centre in Romania. Int. J. Equity Health.

[21] Chambers D.W., Zitterkopf J.G. (2019). How people make decisions about whether or not to seek orthodontic care: Upstream in the treatment chain. Am. J. Orthod. Dentofac. Orthop..

[22] Allen E.M., Call K.T., Beebe T.J., McAlpine D.D., Johnson P.J. (2017). Barriers to Care and Health Care Utilization Among the Publicly Insured. Med. Care.

[23] Mathew R., Sathasivam H.P., Mohamednor L., Yugaraj P. (2023). Knowledge, attitude and practice of patients towards orthodontic treatment. BMC Oral Health.

[24] Almoammar S., Asiri E., Althogbi S.I., Saad R., Al Shahrani A., Hassan N., Alyami B. (2018). Knowledge attitude of general population towards orthodontic treatment in Aseer region Kingdom of Saudi Arabia World. J. Dent..

[25] Alharbi R., Taju W. (2024). Factors Influencing the Decision Process within Seeking Orthodontic Care among the Saudi Population: A Cross-sectional Survey. Open Dent. J..

[26] Madani S.M., Eslamipour F., Sadeghian S., Tahani B. (2023). General dentists’ awareness of orthodontic treatment needs of patients and their referral practices. Dent. Res. J..

[27] Alrejaye N.S., Alnasser L.A., Alsuliman A.F., Alomran D.K., Alshehri H.H., Almalki M.M., Alenazi S.S., Bushnak I.A., Abolfotouh M.A. (2023). Physicians’ Examination and Referral Practices on Orthodontic Problems Among 6-12-Year-Old Children in Saudi Arabia. Clin. Cosmet. Investig. Dent..

[28] Koufatzidou M., Koletsi D., Basdeki E.I., Pandis N., Polychronopoulou A. (2019). Pediatricians’ awareness on orthodontic problems and related conditions-a national survey. Prog. Orthod..

[29] Lin F., Ren M., Yao L., He Y., Guo J., Ye Q. (2016). Psychosocial impact of dental esthetics regulates motivation to seek orthodontic treatment. Am. J. Orthod. Dentofac. Orthop..

