# Supplementary material for: Exploring Parental Perceptions and Barriers to Early Orthodontic Treatment in Children: A Mixed-Methods Study
Source: Healthcare (Basel). 2026 Jan 11;14(2):180. doi: 10.3390/healthcare14020180 (PMC12841336; doi:10.3390/healthcare14020180)
Supplement: Supplementary file 1 [file healthcare-14-00180-s001.zip › healthcare-4071292-supplementary.pdf]

Annexure A

Parental perception of barriers to early orthodontic care during the mixed dentition stage among children in Saudi Arabia

Dear Sir/Madam,

You are invited to participate in an online survey (QR code provided) on our research (Parental perception of barriers to early orthodontic care during the mixed dentition stage among children in Saudi Arabia).

PARTICIPATION: Your participation in this survey is voluntarily; you may refuse to take part in the research or exit the survey at any time without penalty. You are free to decline to answer any particular question you do not wish to answer for any reason. The survey is anonymous as we don't take your name, all data that is collected will be treated with complete confidentiality

CONFIDENTIALITY: Your survey answers will be sent to a link at Google Forms where data will be stored in a password protected electronic format. Google Forms does not collect identifying information such as your name, email address or IP address Therefore, your responses will remain anonymous. No one will be able to identify you or your answers, and no one will know whether or not you participated in the study.

CONTACT: If you have any question regarding the research, please feel free to contact the principal investigator, [redacted] at Phone number #[redacted].

By clicking on the "I agree" button, you acknowledge that: You have read and understood the information provided to you; You voluntarily agree and give consent to be a participant in this study, and you know that you can refuse to answer the questions and/or withdraw from the research at any time, without having to specify the reasons.

o I agree

Thank you in advance for your cooperation.

1. What is your relationship with the child? *

o Mother

o Father

2. How old are you? *

o 18-29 years (<30 years)

o 30-40 years

o > 40 years

3. What is your highest level of education? *

o Primary school or less

o Secondary or high school

o College/University degree

4. **What is your Household monthly income (in Saudi riyals) ?**

o 5000 or less

o 5000 - 15000

o More than 15000

5. **Number of own children ?**

o 1

o 2

o 3 or more

6. **Do you have any Personal History of orthodontic treatment ?**

o Yes

o No

7. **Do you think that your child's teeth would ever have a significant impact on his/her personality?**

o Yes

o No

8. **Do you think your child has any problems with the Teeth alignment/position or skeletal problems?**

o Yes

o No

9. **If yes, to Q8, what problem is it? [Choose one or more answers. [✓]**

- Spacing between teeth
- Crowded anterior teeth
- Protruded upper teeth
- Extra teeth
- Missing teeth
- Incorrect teeth position
- Facial Asymmetry
- Protruding mandible
- Protruded upper Jaw
- Other: __________

10. **Have You ever Consulted a Dentist Or Orthodontist Seeking Orthodontic Evaluation for your child?**

o Yes

o No

11. **If YES to Q10, Did Your Child Receive the Required Orthodontic Treatment?**

o Yes

o No

12. **What are the barriers or obstacles you face in obtaining orthodontic care for your children? [Choose one or more answers. [✓]**

- Dental fear/Anxiety
- Treatment cost
- Lack of Dental insurance coverage
- Lack of knowledge/awareness
- Age (too young)
- Unaesthetic appearance
- Long Treatment duration
- Long waiting list for appointments
- Other: __________
